# Quality of care for people with differences of sex development (DSD) in Germany

**DOI:** 10.1186/s13023-024-03467-5

**Published:** 2024-12-09

**Authors:** Maike Schnoor, Andreas Heidenreich, Martina Jürgensen, Ulla Döhnert, Olaf Hiort, Alexander Katalinic

**Affiliations:** 1 Institute for Social Medicine and Epidemiology, University of Lübeck, Lübeck, Germany; 2 Department of Pediatrics and Adolescent Medicine, Section for Pediatric Endocrinology and Diabetology, University Hospital Schleswig-Holstein, Lübeck Campus, Germany

**Keywords:** Differences of sex development, DSD, Patient-related registry, Health services research

## Abstract

**Background:**

People with "Differences of Sex Development" (DSD) require comprehensive, specialised, and individualised medical and psychological care. This is often perceived as inadequate by those affected. Therefore, the German Federal Ministry of Health funded the project DSDCare which aimed to improve the quality of care for people with DSD over the lifespan in Germany.

**Method:**

Indicators of structural, process, and outcome quality were defined, which are used to evaluate the quality of care. The indicators of structural quality are collected once a year from ten participating centres. Based on the "Open Source Registry System for Rare Diseases (OSSE)", a DSD-specific registry (DSDReg) was developed, in which patient-related care data are recorded in order to be able to assess the process and outcome quality. Furthermore, patient-reported outcomes are collected directly from the patients by means of questionnaires. The data are reported back to the participating centres in an annual benchmarking.

**Results:**

Twenty-five indicators of structural quality were defined, twelve indicators of process quality and ten of outcome quality. A total of 477 patients were registered in DSDReg in the period from May 2021 till October 2022. The mean age is 16 years; the most common diagnosis groups are 46,XY DSD (34.8%), followed by 46,XX DSD (33.3%) and chromosomal DSD (27.5%). Patient numbers vary across centres from N = 10 to N = 131. Questionnaires are available from 316 (66.2%) affected individuals, including 122 from adults, 120 from children or adolescents with DSD, and 191 from parents. Preliminary results show heterogeneity between centres in both data quality and quality of care.

**Conclusions:**

The DSDReg is well established in the DSDCare project as a quality assurance tool with continuously increasing recruitment figures. The implemented quality indicators are applicable, enable a comparison between the participating centres and will foreseeably lead to an improvement of the care of patients with DSD. A long-term continuation of the registry after the end of the initial study period is therefore indicated.

## Background

"Differences of sex development" (DSD) refer to congenital incongruences of chromosomal, gonadal, and phenotypic sexes [1]. DSD is thus an umbrella term for many very different conditions; in some cases, no genetic diagnosis can be made at all. The incidence of occurrence of DSD is estimated at 0.018% to 3.8% of all live births, depending on the definition and the conditions included [2]. DSD thus represent "Rare conditions" [3]. From a medical point of view, there is an acute need for medical therapy only in a few differences of sex development, e.g. in the classical form of congenital adrenal hyperplasia (CAH). Nevertheless, people with DSD require comprehensive, highly specialised and individualised long-term medical care. This includes diagnosis, education and information about possible outcomes, e.g. on pubertal development, fertility, sexuality or tumour risk of gonads, and psychosocial and psychosexual issues [1, 4, 5]. Therefore, support by psychosocial specialists and peer counselling plays an elementary role from the very beginning [6]. In addition, patients must be informed in detail about pros and cons of all therapy options (e.g. hormone therapies, surgery, or no therapy) and recommended check-ups to enable shared decision-making on procedures. However, due to the fragmentation of the care landscape in Germany and a need for more evidence, this comprehensive care for people with DSD is difficult and often perceived as insufficient by those affected [7]. In their statements of 2012 and 2015, respectively, both the German Ethics Council [8] as well as the German Medical Association [9] demanded improvement in care pathways concerning diagnosis, access to treatment options, multidisciplinary care across the lifespan, psychosocial counselling, including peer counselling, and the establishment of special care and reference centres. These demands have been incorporated into the recommendations of the clinical practice guideline "Variants of Sex Development", published in 2016 [10].

From May 2020 to August 2023, the German Federal Ministry of Health has funded the project "Standardised healthcare -center-centered care of DSD over the lifespan", **DSDCare**, (funding code 2519FSB503), which aims to improve the medical care of people with DSD in Germany by implementing the guideline recommendations. In addition to developing a concept for quality-improving interdisciplinary and multi-professional care of people with DSD, the project harmonises hormone and genetic diagnostics, develops further training for service providers and education courses for those affected, and establish a web-based information portal (info.dsdcare.de, German only) for those providing care outside the participating centres. Furthermore, the DSDReg registry was established to evaluate standardised care data from patients. This makes it possible to map the quality of care using quality indicators (QI) and to report it back to the participating centres in the form of benchmarking. Benchmarking is considered an important instrument of quality assurance in the health care system. Comparisons between service providers enable them to learn from each other and thus to monitor control and improve the care of those being treated in the long term [11]. In the following, we present the first data from the DSDCare project spanning the period from May 2021 to October 2022.

## Methods

### Study population

Since May 1, 2021, patients with DSD conditions, according to the 2005 Chicago Consensus Conference, have been recruited in ten DSD centres participating in DSDCare [12] as well as patients without a diagnosis but with clinical symptoms of a difference of sex development according to the German guideline [10]. Exclusion criteria have been presence of acquired hypogonadotropic hypogonadism and constitutional delay of pubertal development. Inclusion is based on written informed consent from the patients or their legal guardians in the case of minors. A positive ethics vote was given by the ethics committee of the University of Lübeck (20–322). The study is registered in the German Register of Clinical Studies (DRKS00022521).

### Development and collection of quality indicators

Following Donabedian [13, 14], the quality of care in the DSDCare project is divided into the dimensions of structural, process, and outcome quality. The corresponding quality indicators (QIs) were developed based on the German guideline [10] and evaluated in a comprehensive literature review. These were supplemented by the results of a written qualitative survey of medical and psychosocial professionals involved in the project, patient advocacy groups (AGS-Eltern- und Patienteninitiative e.V., Intergeschlechtliche Menschen e.V.) as well as patients and parents recruited in the DSD consultations. Subsequently, the QI were approved by the project's steering group and reformulated into corresponding parameters to be collected. Since 2020 the structural quality items are collected annually from the ten centres participating in DSDCare. Since May 1, 2021 patient-related process and outcome quality parameters have been continuously entered into DSDReg, which is based on the "Open Source Registry System for Rare Diseases (OSSE)", funded by the German Federal Ministry of Health [15]. Study participants and, in case of minors, their legal guardians participating in DSDCare receive a questionnaire to collect PROMs (Patient-Reported Outcome Measures) and PREMs (Patient-Reported Experience Measures) at inclusion and 6 as well as 12 months later.

### Survey instruments for the recording of PROMs and PREMs

Standardised, validated survey instruments are used to collect patient-reported outcomes. Treatment satisfaction is assessed using CHC-SUN "Child Health Care- Satisfaction, Utilization and Needs". [16, 17] by parents and adults, respectively, and YHC-SUN "Youth Health Care- Satisfaction, Utilization and Needs" [18] of adolescents aged 13 years and older. Quality of life is assessed according to the age group using either the KIDDY KINDL- external assessment questionnaire [19], the KIDSSCREEN 52 questionnaire [20, 21] or the WHOQOL-BREF [22]. From age eight, participants are asked to answer additional questions about shame and stigmatisation. The age group of 13 to 21 also receives a questionnaire on skills related to the transition from pediatric to adult medicine [23].

### Data evaluation

The statistical analysis was descriptive, stratified by centre of care for benchmarking. Continuous data are presented using the mean and standard deviation or median and interquartile range (IQR), categorical variables in absolute and relative frequencies. The validated questionnaires were analysed according to the respective evaluation manual. For the analysis of process and outcome quality, data of all patients were considered who were included in the DSDCare study and entered into the registry until October 31, 2022.

## Results

### Study population

**Fig. 1:**
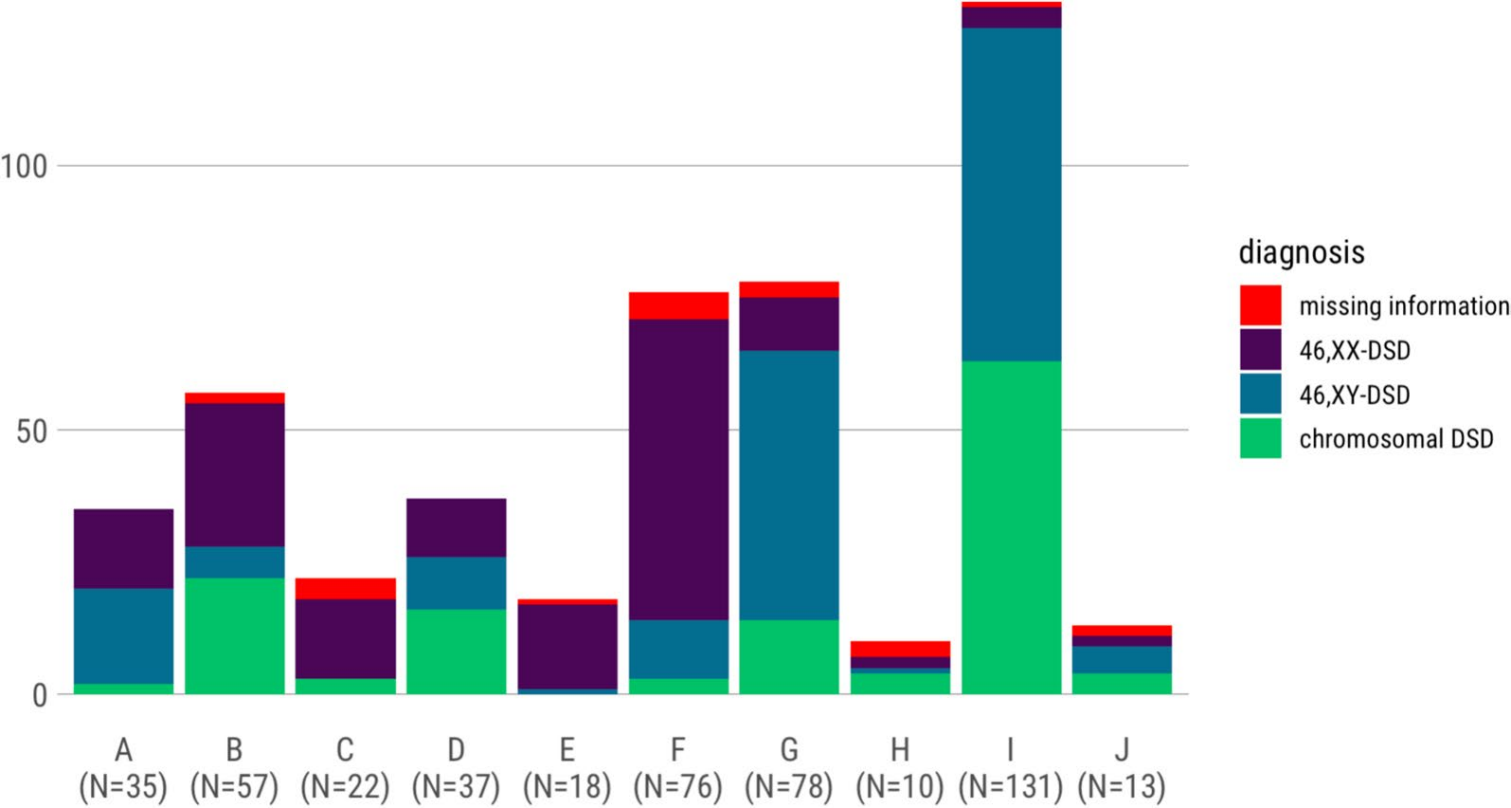
Diagnoses according to DSD classification by centres

From 05/01/2021 to 10/31/2022, 477 patients were included in DSDReg. The mean age was 16 years (IQR 10). The initial entry of sex in the birth registry was female for 56.6% (N = 270) of patients, male for 39.6% (N = 189), diverse for 0.4% (N = 2), and open for 1.9% (N = 9). For four participants (0.8%), the sex of the initial entry was unknown, and for three (0.6%), the record was missing from the registry. The most common diagnostic group was 46,XY DSD (34.8%), followed by 46,XX DSD (33.3%) and chromosomal DSD (27.5%). The number of patients in the centres varies between N = 10 and N = 131 (Fig. 1).

### Structural quality

**Fig. 2:**
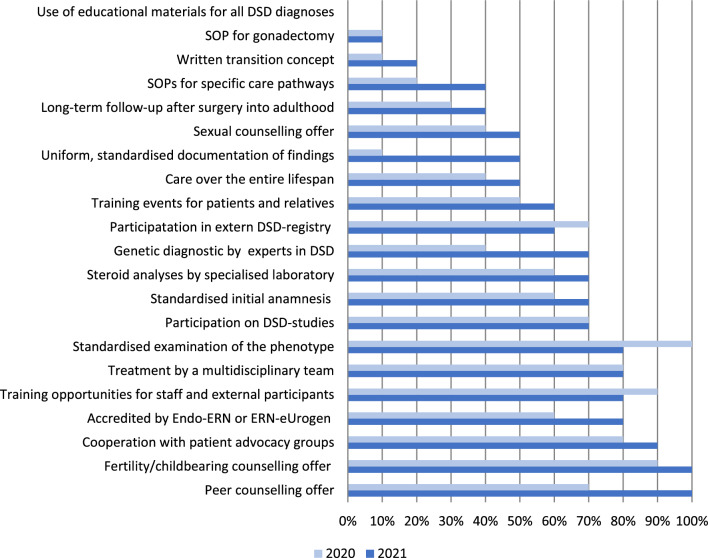
Proportion of centres who reached indicator of structural quality

25 QIs on structural quality were approved, 21 of which were already collected in 2020. For 14 of these 21 QIs, the number of centres fulfilling the respective QI has increased over the course of the DSDCare project, and for four QIs, it has remained the same (Fig. 2).

While some quality criteria were fulfilled by all centres in 2021 (offering peer counselling, offering fertility or infertility counselling), others are still barely established (e.g., the existence of a concept for transition to adult medicine) or not established at all (educational materials for the entire DSD spectrum). The number of structural QIs fulfilled per centre varies between six and fifteen in 2020 and six and eighteen in 2021.

### Process quality

The implemented indicators of process quality include (age-appropriate) comprehensive information of the affected persons and, if necessary, their parents, the performance of hormonal and genetic diagnostics and molecular-cytogenetic diagnostics for the diagnostic groups 46,XY DSD and 46,XX DSD. Hormonal values should be evaluated specifically in the context of DSD. Further QI refer to the implementation of a case conference (1 × per year per patient*), the offer of psychological counselling, sexual counselling, fertility counselling and peer counselling, and the provision of contact with support groups. Psychological counselling, peer counselling and a case conference should have taken place before performing of surgery.

The centers differ significantly in terms of data quality and the extent to which QIs are met. For example, the proportion of patients with a genetic diagnosis varies by centre from 37 to 94% (missing values 0% to 45%) (Table 1).

**Table 1:** Selected process quality indicators and their fulfilment per centre

Center (Case number)	A (N = 35)	B (N = 57)	C (N = 22)	D (N = 37)	E (N = 18)	F (N = 76)	G (N = 78)	H (N = 10)	I (N = 131)	J (N = 13)
*Psychosocial counselling provided*										
yes	2 (5.7%)	44 (77.2%)	1 (4.5%)	28 (75.7%)	—	72 (94.7%)	50 (64.1%)	6 (60.0%)	127 (96.9%)	5 (38.5%)
no	4 (11.4%)	2 (3.5%)	—	1 (2.7%)	—	—	12 (15.4%)	2 (20.0%)	2 (1.5%)	8 (61.5%)
missing values	29 (82.9%)	11 (19.3%)	21 (95.5%)	8 (21.6%)	18 (100%)	4 (5.3%)	16 (20.5%)	2 (20.,0%)	2 (1.5%)	—
*Hormonal diagnostisc performed*										
yes	7 (20.0%)	38 (66.7%)	22 (100%)	37 (100%)	—	64 (84.2%)	58 (74.4%)	9 (90.0%)	126 (96.2%)	9 (69.2%)
no	6 (17.1%)	16 (28.1%)	—	—	—	3 (3.9%)	13 (16.7%)	1 (10.0%)	3 (2.3%)	3 (23.1%)
missing values	22 (62.9%)	3 (5.3%)	—	—	18 (100%)	9 (11.8%)	7 (9.0%)	—	2 (1.5%)	1 (7.7%)
*Genetic diagnostics performed*										
yes	13 (37.1%)	50 (87.7%)	12 (54.5%)	29 (78.4%)	17 (94.4%)	56 (73.7%)	53 (67.9%)	9 (90.0%)	122 (93.1%)	7 (53.8%)
no	21 (0.6%)	5 (8.8%)	—	7 (18.9%)	1 (5.6%)	1 (1.3%)	17 (21.8%)	—	5 (3.8%)	4 (30.8%)
missing values	1 (2.9%)	2 (3.5%)	10 (45.5%)	1 (2.7%)	—	19 (0,3%)	8 (10.3%)	1 (10.0%)	4 (3.1%)	2 (15.4%)

For the QI "offer of psychological counselling," the proportion of missing values is particularly high; one centre did not provide any information on the QI. For the other nine centres, the proportion of missing values ranges from 0% to 95.5%. Some process quality indicators, such as surgery preparation, affect only some of the study participants, so that comparisons between centres are not useful at this stage due to the low number of cases overall (n = 48, including gonadopexy and inguinal hernia surgery).

### Outcome quality

The outcome quality includes indicators that are recorded by the centre (surgical complications) and by the participants or their guardians through questionnaires (treatment satisfaction, quality of life, transition competence, and acceptance of the own body).

**Fig. 3:**
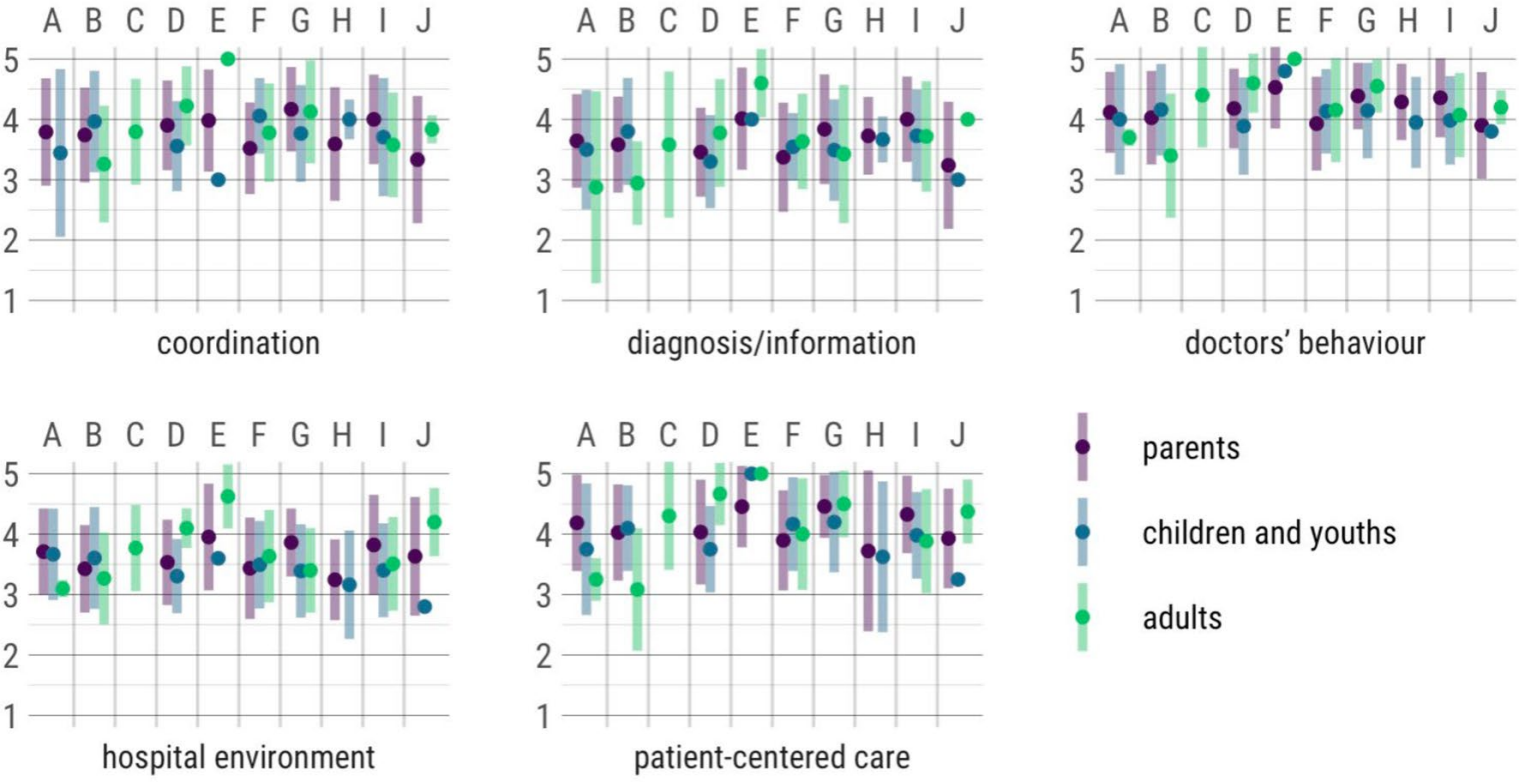
Mean satisfaction by dimensions and centres

Data from questionnaires are available from 316 (66.2%) participants, including 122 from adults with DSD, 120 from children or adolescents, and 191 from parents. However, due to the small number of cases per survey group (adults, children/adolescents, parents) and centre, it is not yet possible to make reliable statements on differences in the quality of outcomes between the centres. First evaluations regarding treatment satisfaction show a relatively high satisfaction in all five dimensions ("diagnosis", "coordination and circumstances", "organisation", "patient-centred care", "behaviour and attitude of the physicians") of the CHC-SUN or YHC-SUN (Fig. 3).

Large variances are found in transition competence in the age group of 13- to 21-year-olds (N = 242). Complete information on transition skills is provided from 61% (N = 148) of participants in this age group. The total score ranges from 16 to 90 out of 100 achievable points (Table 2). Even within the three dimensions of "work environment," "disease-related knowledge," and "care competence," scores vary considerably (work environment: 0 to 78 points; disease-related knowledge: 44 to 100 points; care competence 8 to 92 points). However, centres that score very well or poorly include data from only a few individuals.

**Table 2:** Transition skills by dimension and centre (mean (standard deviation))

Centre	N	Working Environment	Disease-related knowledge	Care competence	Overall
A	3	77.78 (18.14)	70.37 (13.86)	63.89 (27.50)	70.00 (14.14)
B	16	64.58 (29.45)	58.33 (29.00)	46.88 (25.02)	55.63 (25.02)
C	1	66.67 (–)	100 (–)	91.67 (–)	86.67 (–)
D	15	67.41 (36.62)	68.15 (36.71)	45.56 (19.69)	57.62 (26.65)
E	1	0.0 (–)	44.44 (0)	8.33 (–)	16.67 (–)
F	37	67.87 (36.51)	74.77 (28.14)	62.39 (25.46)	67.75 (26.09)
G	14	52.38 (38.82)	62.70 (32.42)	50.00 (23.36)	54.52 (24.90)
H	4	47.22 (41.85)	62.96 (37.77)	11.11 (10.39)	33.33 (21.77)
I	56	54.56 (34.90)	63.69 (28.68)	51.93 (18.37)	56.25 (24.87)
J	1	100 (–)	100 (–)	75.0 (–)	90.0 (–)

## Discussion

Despite numerous statements and recommendations for action, the care and equality of people with DSD still needs to be further improved and monitored. In order to avoid discrimination and misprovision the legislature amended the Personal Status Act in 2018 [24]. Since then, it has been possible to specify “diverse” as sex in addition to female and male, or to specify no sex. Since 2021 the new Act on the "Protection of Children with Differences of Sex Development" regulates surgical interventions on minors with DSD, which serve the purpose of gender reassignment to the female or male sex [25]. These can only be carried out when the child can make a self-determined decision. Otherwise, a family court must be involved.

The anticipated improvement in medical and psychosocial care requires continuous monitoring. Patient-based registries systematically record the reality of care and its changes over time, with the essential aim of evaluating care [26]. Comparisons of actual care with quality indicators and existing guidelines provide information about the quality of treatment in terms of overuse, underuse and misuse. However, registries for rare conditions, such as the DSDReg, face unique challenges due to the small number of cases and the heterogeneity of diagnoses [27].

With regard to DSD diagnoses, several international registries, such as the I-DSD Registry [28] or the I-CAH registry [29], have been established. Also, since 2018, the European Reference Networks for Rare Endocrine Diseases (ENDO-ERN) has been using EuRRECa registries (European Rare Endocrine Disease Registries) [30]. However, the existing registries are not suitable to adequately reflect the quality of care at the national level and thus allow a long-term improvement in the care of people with DSD in Germany. For example, the I-DSD registry does not collect outcome-quality data. The I-CAH, on the other hand, is limited to the diagnosis of CAH only. The EuRRECa registries collect generic but no DSD-specific data on outcome quality. In this respect, an instrument that addresses the care of people with DSD nationally and in all three quality dimensions has been lacking. In particular, including patient-reported outcomes (treatment satisfaction, quality of life, transition skills) and patient reported experience measures, which can be entered electronically directly by study participants into the registry, is an essential component of the DSDReg. While in other countries such as Sweden, Norway, Denmark, the USA or The Netherlands, PROMs are components of the national registries, in Germany, there are no national strategy or guidelines for the collection of PROMs [31]. However, their inclusion is now called for in the memorandum "Registries for Health Services Research", as this reflects the perspective of those affected and therefore increases the relevance and informative value of registries and the validity of the results [26]. With this in mind, the German Federal Ministry of Education and Research announced the funding initiative "Establishment of model patient-based registries for health services research" at the end of 2016. At least three of the six funded projects include patient-reported outcome by using an APP to enter data by the participants [32, 33] or a Web link to answer a questionnaire online [34]. However, the projects are still in planning or implementation, data on acceptability or feasibility are not available.

The results of the first benchmarking within DSDCare show that the QIs developed in the DSDCare project are applicable and implementable. They allow a direct and fair comparison of the quality of care at different levels between the centres. The first analyses show that all centres still have room for improvement regarding the fulfilment of the QI. With regard to the individual quality parameters of structural quality, the number of centres that met these increased for almost all of them in 2021. The observed decrease in the QI “standardised examination of phenotype” is possibly due to the fact that it was re-specified after the first survey, in the sense that a written standard must be available.

However, SOPs on guideline-compliant care pathways have been developed within the project duration of DSDCare and can now be implemented, which is expected to lead to an initial improvement in the quality of care. Future evaluations will show to what extent this is successful. The currently observed heterogeneity of the centres may be due to the different focus on paediatrics or adult medicine as well as a speciality- or diagnosis-specific focus of care, the number of patients in the centre, and to the quality of the data, which in some cases still needs to be improved. Furthermore, for an assessment of quality of care, for the indicators of process and outcome quality, threshold values should be developed, above which one speaks of good quality. This also applies to data on surgery, which represent an essential component in improving the quality of care.

## Conclusion

The DSDReg is well established as a quality assurance tool in the DSDCare project with continuously increasing recruitment numbers. By continuation of the registry, supported by regular evaluation, the number of patients in the registry can be increased, and the data quality further enhanced. This would allow reliable statements to be made on the quality of and differences in care, over time, also with regard to the treatment of the various diagnostic groups. Furthermore, in the long term, it would be conceivable to develop these DSD-specific QIs together with the criteria for B centres of the National Action League for People with Rare Diseases (NAMSE) [35] for certification as a DSD centre, which would then ensure quality-assured, guideline-based care for people with DSD. This makes the DSDReg an important quality management tool for caring for people with DSD in Germany.

The quality indicators are subject to regular review and further development. The current set of quality indicators can be viewed under: https://research.uni-luebeck.de/de/projects/dsdcare-differences-of-sex-development and are published by Juergensen et al. [36].

## Data Availability

The data that support the findings of the study are not publicly available due privacy issues of participating centres.
