# Correction to: Quality of care for people with differences of sex development (DSD) in Germany

**DOI:** 10.1186/s13023-025-03638-y

**Published:** 2025-03-12

**Authors:** Maike Schnoor, Andreas Heidenreich, Martina Jürgensen, Ulla Döhnert, Olaf Hiort, Alexander Katalinic

**Affiliations:** 1 Institute for Social Medicine and Epidemiology, University of Lübeck, Lübeck, Germany; 2 Department of Pediatrics and Adolescent Medicine, Section for Pediatric Endocrinology and Diabetology, University Hospital Schleswig-Holstein, Lübeck Campus, Germany

Following publication of the original article [1], we have been notified that first and last names of authors were switched. Also, legend to Fig. 3 was published incorrectly.

They are now as follows:

Schnoor Maike1*, Heidenreich Andreas1, Jürgensen Martina2, Döhnert Ulla2, Hiort Olaf2 and Katalinic Alexander1

**Figure 3** Diagnoses according to DSD classifcation by centres

They should be as follows:

Maike Schnoor 1*, Andreas Heidenreich 1, Martina Jürgensen 2, Ulla Döhnert 2, Olaf Hiort 2 and Alexander Katalinic 1

**Figure 3** Mean satisfaction by dimensions and centres

The original article was updated.

## References

[1] Maike et al. (2024) Quality of care for people with differences of sex development (DSD) in Germany (2024). 19:460 10.1186/s13023-024-03467-5 10.1186/s13023-024-03467-5 PMC11626742 39654033

